# Genomics in neurodevelopmental disorders: an avenue to personalized medicine

**DOI:** 10.1038/s12276-018-0129-7

**Published:** 2018-08-07

**Authors:** Dora C. Tărlungeanu, Gaia Novarino

**Affiliations:** Institute of Science and Technology (IST) Austria, Klosterneuburg, Austria

## Abstract

Despite the remarkable number of scientific breakthroughs of the last 100 years, the treatment of neurodevelopmental disorders (e.g., autism spectrum disorder, intellectual disability) remains a great challenge. Recent advancements in genomics, such as whole-exome or whole-genome sequencing, have enabled scientists to identify numerous mutations underlying neurodevelopmental disorders. Given the few hundred risk genes that have been discovered, the etiological variability and the heterogeneous clinical presentation, the need for genotype—along with phenotype-based diagnosis of individual patients has become a requisite. In this review we look at recent advancements in genomic analysis and their translation into clinical practice.

## Neurodevelopmental disorders: Translating genomic advances into clinical practice

The identification of genetic mutations associated with neurodevelopmental disorders (NDDs) along with routine diagnosis based on patients’ characteristics is aiding the delivery of personalized therapies. Dora Tarlungeanu and Gaia Novarino at the Institute of Science and Technology in Klosterneuburg, Austria, review recent advances in genetic technologies, such as whole exome sequencing, that can lead to early intervention, guide choice of treatment and prompt genetic counseling. Introducing the mutations associated with NDDs into model organisms or stem cells is revealing some of the mechanisms underlying NDDs and enabling the evaluation of novel therapeutic strategies that target core symptoms of the disorders. To accelerate the implementation of individualized treatments for NDD the authors highlight the need to adopt interdisciplinary research approaches and to keep clinical staff updated on the latest findings in NDD genetics.

## Introduction

The past decade has seen a rapid development of precise technological and methodological advancements in genetics and genomics, thus allowing an unprecedented identification of mutations that are involved in complex neurodevelopmental conditions. Neurodevelopmental disorders (NDDs) affect more than 3% of children worldwide and can be attributed to mutations at over 1000 loci^1^.

Understanding the etiology of NDDs faces many challenges that range from delineating the heritable genetic components to defining individual factors that predispose to NDD risk and identifying the precise mechanisms through which these factors together lead to the disorder^2^. In addition, the clinical heterogeneity of NDDs make diagnosing a lengthy and costly process, complicating the quest for personalized medicine. However, the identification of bona fide genetic risk factors and the use of functional genomics to progress from mutation to phenotype represent a solid foundation for the development of individualized therapeutic approaches. In this review, we begin by mentioning some features of these disorders and continue by emphasizing the importance of genomics in determining the etiology of NDDs. We then describe advantages and limitations in the use of animal or stem cell models to study patient-specific genetic mutations. Finally, we discuss successful examples of translational research creating an evidence-based framework of how personalized medicine can advance the treatment of NDDs.

## Neurodevelopmental disorders

NDDs are a group of early onset neurological disorders, including autism spectrum disorders (ASD), intellectual disability (ID) and language disorders among others. ASDs are characterized by early dysfunction in social interactions, communication deficits, and the presence of repetitive and restricted behaviors^3^. ASDs, with an estimated prevalence of 1 in 68 births^3^, represent an issue of public concern. Typically, ASDs have been classified into syndromic—Rett syndrome (RS)^4^, Fragile X syndrome (FXS)^5^, and tuberous sclerosis (TSC)^6^—and nonsyndromic. Evidence suggests that the causes involve both genetic and environmental factors^7^. Patients diagnosed with ASD often present with other comorbidities such as intellectual disability (ID)^8^, epilepsy^9^, and motor abnormalities^10^. Intellectual disability affects ~1.5–2% of the Western population^11^. The severe forms of ID are thought to have a genetic origin, but in at least 50% of cases, the cause remains elusive. Over the past years many autosomal or X-linked mental retardation genes have been identified^12^, with *FMR1* (FXS) being one of the most common inherited monogenic causes of ID and ASD in male patients^13^. The core features of ASD and ID often coexist with recurrent seizures or epilepsy. Epileptic seizures are due to abnormal neuronal activity such as excessive excitation or hypersynchronization, which can occur as a result of developmental defects or due to brain insults (e.g., trauma, stress, etc.) later on in life. With over 65 million people affected worldwide, epilepsy is the most common, chronic neurological disorder^14^. Although in many cases seizures can be controlled by existing anti-epileptic drugs, the treatment gap is still large^15^. Genetic underpinnings for epilepsies have been long recognized and over the past 20 years a significant number of epilepsy-risk genes have been identified^16,17^.

## The genetics of NDDs

On average, a newborn acquires between 50 and 100 new genetic variants, resulting in 0.86 new amino acid-altering mutations (i.e., de novo mutations) per individual^18^. Given such a high individual variability, a plethora of variants associated with NDDs have been found in hundreds of different genes, ranging from single nucleotide changes (single nucleotide variants (SNV)) to loss or gain of up to thousands of nucleotides (copy number variants (CNV)).

**Fig. 1:**
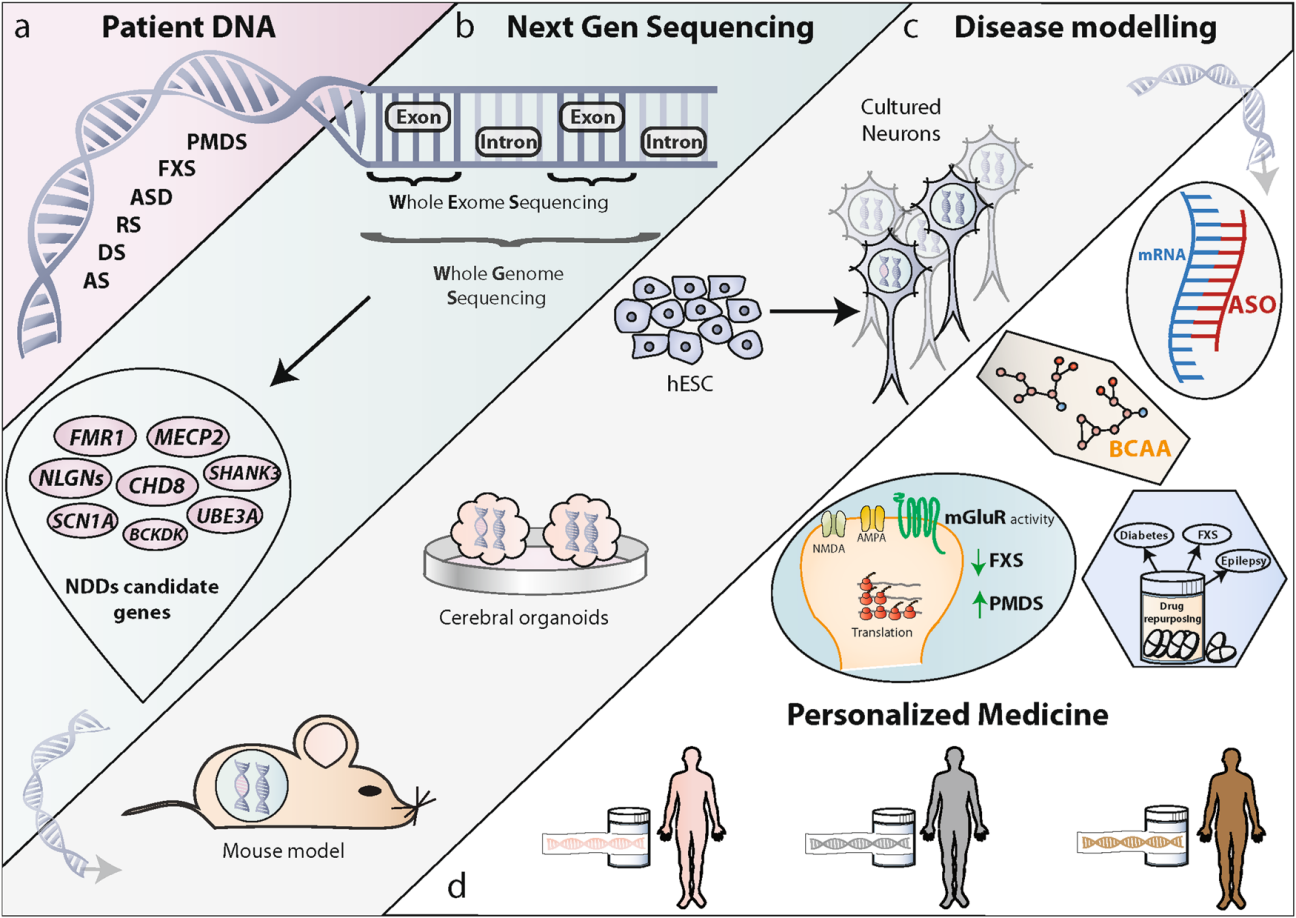
Genomic sequencing guides the way from patient DNA to personalized medicine. **a** DNA from patients diagnosed with NDDs used for sequencing; FXS Fragile X Syndrome, RS Rett Syndrome, PMDS Phelan McDermid Syndrome, DS Dravet Syndrome, AS Angelman Syndrome, ASD autism spectrum disorder. **b** Next-generation sequencing can be used to decipher the genetic code within exons (dark blue section—whole-exon sequencing) or throughout the entire genome (dark and light blue section—whole-genome sequencing). Mutations are identified in a series of genes with predisposition to NDDs (pink ovals). **c** The mutations are regenerated in models (mice, organoids, or hESC-derived neurons) in order to understand their underlying mechanism. **d** Disease modeling reveals targets that enable the implementation of personalized medicine. ASO (antisense oligonucleotides—gray panel) and BCAA (branched chain amino acids—beige panel) are two examples of personalized therapies probed in mouse models. mGLUR (metabotropic glutamate receptor) activity (green panel) needs to be decreased in FXS and increased in PMDS. Drug repurposing (blue panel) enables the usage of the same drug for different diseases due to novel mechanisms identified

Sequencing of the human and other mammalian genomes has provided an important set of tools to start understanding the human genetic variation. The first steps to elucidate the genetic heterogeneity of NDDs were done by using karyotyping or fluorescence in situ hybridization (FISH). As the need for more accurate detection of nucleotide variations in the context of developmental disabilities grew, chromosome microarray (CMA) technology was developed and rapidly implemented as part of first-line evaluation for children with a NDD^19,20^. CMA set the stage for genetic variation detection, but the advent of whole-genome and whole-exome sequencing (WGS and WES) led to the identification of many inherited and de novo germline variants that significantly contribute to total NDD risk^21–24^ (Fig. 1a, b). In the case of ASD for instance, it is estimated that rare genetic mutations, both de novo and inherited, are causal in ~11% of simplex cases^25^. Similarly, common inherited genetic variants contribute substantially to ASD risk (49%); however, the individual common genetic variant liability is lower than that for rare genetic mutations^26^. Likewise, in the case of epilepsy, rare CNVs have been shown to explain ~3% of individuals suffering from idiopathic generalized epilepsy^27^. In addition, ~300 de novo mutations were identified in patients suffering from epileptic encephalopathies. These mutations emphasize the convergence on specific biological pathways due to their enrichment in certain gene sets including genes regulated by the fragile X protein^28^. Finally, germline mutations do not explain all NDD cases, indicating that other genetic defects also come into play. For example, postzygotic (i.e., somatic) mutations explain a significant proportion of NDD cases^29–31^. Along with the previously identified CNVs, such mutations have meaningful implications for risk prediction, diagnosis and patient management^32^.

Thus, the abovementioned technologies represent powerful tools for the molecular genetic dissection of patients affected by NDDs. Their introduction into clinical practice and association with routine phenotype-driven diagnosis holds promise for personalized diagnosis and therapy of NDDs.

## The promise of genetics

The early occurrence of genetic glitches and the relatively late onset of symptoms that enable the diagnosis of NDDs, represent a major pitfall in identifying the cause and delivering the right kind of therapy. To complicate things further, for most NDDs, therapies hinge largely on behavioral or educational interventions^33^ and on treating associated rather than core symptoms of the disorder. Thus, for the majority of people with NDDs, the outcomes are poor or very poor in adulthood^34^. Given such challenges, we must ask how genetics may contribute to their improvement.

First and foremost, genetic testing can lead to active monitoring and early intervention, even before the onset of the disorder. Furthermore, knowing the genetic cause of a disorder may reveal the role of a specific biological pathway in its onset. Thus, targeted pharmacological interventions could be made with already existing drugs. Studies reported that 55% of 187 genetic findings led to changes in clinical management^35^ and 10 out of 118 probands undergoing WES benefited from a revised diagnosis and clinical assessment^36^. Similarly, WES with targeted gene analysis (e.g., *SCN1A*) influenced decisions on antiepileptic drug selection and reconsideration of surgical interventions^37^. Lastly, since NDDs are associated with cognitive and behavioral abnormalities, genetic information can guide the choice of behavioral treatment^38^. However, despite these advantages, the multiple guidelines proposing the use of genetic testing for individuals with NDDs are not implemented routinely in clinical practice^20^. This lack of use is either due to scarcity of resources or due to a lack of medical staff prepared to analyze and interpret genetic results. To circumvent this issue, a proper dissemination of up-to-date findings about NDD genetics to clinical staff is desirable. In addition, genetic counseling should inform parents about recurrence risk assessment.

## Modeling NDDs: potentials and limitations

An ideal model of a human disorder is characterized by construct validity (model mimics the genetic insult that causes the disease), face validity (the model’s phenotype resembles that of the human disease), and predictive validity (the model and the patients respond similarly to certain treatments). Several systems (cells, rodents, primates) have been used to generate models of NDDs that can partially reproduce disease features and can be of interest for understanding underlying mechanisms (Fig. 1c).

The most favored model organism, the mouse, has been extensively employed for modeling neurological disorders with a known genetic cause, such as FXS (*Fmr1* KO) (FXS)^39^, Dravet syndrome (DS-*Scn1a* KO)^40^, ASD *Nrxn1a* KO^41^, *Nlgn3* KO^42^, Phelan-McDermid syndrome (PMDS-*Shank 3* KO)^43^ or RS (*Mecp2* KO)^44^. Mice share 95–98% of their genomic information with humans, have a relatively rapid reproduction time, are cost-effective and allow scientists to precisely manipulate their genome in a temporal/spatial specific manner. However, mice also present with important limitations. For example, assessment of higher brain functions, such as language and facial recognition, is difficult in large screens. To overcome some of these limitations, non-human primates can be employed to model complex behavior and higher cortical functions^45^, whereas zebrafish and invertebrates can be used for high-throughput genetic screens^46^.

Alongside animal models, in vitro reprograming of stem cells has enabled the generation and analysis of human neurons. Employing either human embryonic stem cell (hESC)-derived or human induced pluripotent stem cell (hiPSC)-derived neurons, researchers have recapitulated several neuronal synaptic defects for monogenic forms of NDDs such as RS^47^, FXS^48^, Prader-Willi and Angelman syndromes (AS)^49^, PMDS^50^, DS^51^ and Timothy syndrome (TS)^52,53^. The experimental tractability, the ability to model diseases directly from affected individuals and the unlimited source of cells are just some of the advantages of stem cell-based models. Conversely, the high heterogeneity among iPSC clones, the immature identity of neurons differentiated in vitro, the lack of high-order connectivity and the difficulty to model lamination in a 2D system are some of the obvious shortcomings of iPSC-derived disease models. Fortunately, recently several researchers have developed protocols for the generation of 3D cortical organoids (mini-brains/spheroids), providing avenues to study features of cortical lamination and brain development in vitro^54,55^, thus contributing additional tools for studying the mechanisms underlying NDDs^52,56^.

## Bridging the gap between research and the clinic—personalized therapeutic approaches for NDDs

Axiomatically, the biggest advantage of genetic studies is to provide clues about the underlying neurobiology of NDDs and to transition those clues into clinical practice (Fig. 1d).

At present, the available treatments for NDDs consist of a combination of behavioral therapies^57^ and drugs approved for ameliorating comorbidities such as irritability and anxiety, while in many cases the core symptoms of NDDs remain unsolved.

However, the combination of genetics and functional analysis led to the discovery of several molecular pathways involved in NDDs that were targeted to evaluate novel therapeutic strategies. Particularly, inhibiting the mechanistic target of rapamycin (mTOR) rescues physiological, morphological and behavioral abnormalities in mice modeling diseases associated with protein translation defects such as TSC^58^, *PTEN-* associated macrocephaly^59^ or 15q11-13 duplication syndrome^60^. Multiple clinical trials are investigating the pharmacokinetics and pharmacodynamics of rapamaycin and its analogs (sirolimus, everolimus) for treating TSC with associated ASD^61,62^. Likewise, increasing levels of (IGF1) -like growth factor 1 and brain-derived neurotrophic factor via transcriptional modulation improves physiological and behavioral anomalies in RS mouse models^63,64^ and IGF1 administration leads to a higher endurance to social and cognitive testing in patients with RS^65^ or PMDS^66^.

Modulation of the excitation/inhibition ratio by employing antagonists of mGluRs or agonists of GABA A and GABA B receptors, has also been considered as a potential strategy to treat NDDs^67^. However, contrary to what was predicted by a decade of studies in FXS animal models, administration of mavoglurant, an mGluR5 antagonist^68^, or arbaclofen, a GABA B receptor agonist^69,70^, to adolescents and adults with FXS showed no significant improvement in behavioral traits in a randomized, double-blind, placebo-controlled phase 2 trial. Conversely, in a mouse model of PMDS with complete deletion of *Shank3*, researchers reported decreased mGluR5 signaling in the striatum and cortex. Administration of a benzamide derivative resulted in augmentation of mGluR5 activity and rescue of functional and behavioral defects in mice. Thus, pharmacological treatments aimed at increasing mGluR5 activity may represent an option for patients with S*HANK3* mutations^71,72^. The contrasts between mGluR5 activity in FXS and PMDS suggest that the dysfunction leads to distinct phenotypes in different brain regions/genetic backgrounds. Hence, genetically discriminating between different forms of NDDs and identifying the convergence of the common molecular pathways underlying NDD pathophysiology are important goals (Fig. 1d).

Recently, new therapeutic strategies have been designed based on genetic findings. For example, AS is mostly caused by loss-of-function mutations in the maternal allele of the imprinted *UB3A* gene, while the paternal allele is silenced by a long noncoding RNA (*UBE3A* antisense transcript). Using antisense oligonucleotides (ASOs) (Fig. 1d) the paternal allele was unsilenced, thereby restoring the UB3A protein levels and leading to improvement in cognitive deficits in an AS mouse model^73^. Following a similar rationale, ASOs are used for restoring normal levels of MeCP2 and rescuing neurological deficits in mice carrying an extra copy of *Mecp2*^74^. Replacing a defective gene may also be achieved by gene therapy using adeno-associated virus (AAV) vectors^75^. However, making ASOs and AAV amenable to translation into clinical trials is challenging due to their safety, pharmacokinetics and distribution in the brain^76^. In a similar vein, WES of consanguineous families with ASD, ID and epilepsy led to the identification of mutations in the gene *BCKDK* (Branched Chain Ketoacid Dehydrogenase Kinase), encoding an enzyme regulating the catabolism of the branched-chain amino acids (BCAAs). The *Bckdk* mouse model displays an abnormal brain amino acid profile and dietary supplementation with the missing BCAAs reverses certain neurological phenotypes (Fig. 1d). Furthermore, BCAA dietary supplementation in patients led to normalization of plasma BCAA levels, demonstrating the potential of BCAA supplementation as a therapy in patients with *BCKDK* mutations^77,78^.

In addition to identifying new targets for therapy, genetic findings are useful for personalizing existing pharmacotherapy or behavioral interventions. In this sense, WES with targeted gene analysis (e.g., *SCN8A, KCNQ2*) is an effective diagnostic tool for patients with epilepsies as it can influence anti-epileptic drug selection, adverse effect minimization and consideration for surgery, based on each patient’s genetic script^37^. In the case of people with *SHANK3* deletions, they tend to have more advanced receptive communication skills than verbal language ability^79^ and therefore could benefit from assistive communication strategies that may not have been in mind unless the genetic cause of their ASD was known.

Recently, a very common trend uses genetic findings for the application of targeted drug repurposing based on single gene defects (Fig. 1d). Such an approach already shows promise for personalizing therapies for epilepsy cases arising from gain-of-function mutations in ion-channel subunit genes (e.g., *GRIN2A, GRIN2B, SCN8A*). Nonetheless, important barriers remain in order to translate these approaches to non-ion channel epilepsy genes and loss-of-function mutations^80,81^. Likewise, recent observations indicate that metformin, a worldwide first-line therapy for type 2 diabetes, rescues core phenotypes in adult FXS mice due to normalization of ERK signaling, eIF4E phosphorylation and matrix metalloproteinase 9 expression (MMP-9)^82^. Given that the previously mentioned clinical trials with mGluR5 antagonists have failed, metformin represents a new therapeutic avenue for clinical studies involving FXS patients. Administration of oxytocin, which is a peptide usually administered to initiate uterine contractions that also appears to be involved in modulating social behavior, improves ASD-like social deficits in several mouse models^83^ and in a *Shank3*-deficient rat^84^, but the clinical effectiveness of oxytocin on ASD should still be considered tentative due to mixed findings^85^.

## Conclusion

The quick development of novel and efficient sequencing technologies made the identification of genetic causes for a number of NDDs possible. Using these techniques, an underlying genetic cause of many NDD cases can be identified. This progress allows the design of personalized therapeutic strategies and the implementation of genetic counseling. Furthermore, studies employing animal and human cell models carrying specific genetic glitches are underscoring potential novel therapeutic approaches.

In the past few years, potential treatments derived from genetic and functional analysis made it to clinical trials. Although several clinical trials have failed, the treatment of some NDDs seems much closer. Due to the very complex nature of NDDs, interdisciplinary approaches combining genetics, functional genomics, robust biological models and objective measures of response, such as biomarkers^86^, as well as the capability of researchers and clinicians to work side by side, will be essential.

Data for this review was collected by typing the following keywords into PubMed: genomics, genetics, personalized therapy, neurodevelopmental disorders (all in combination with NDDs, ASD, ID).
